# From Near-Optimal Bayesian Integration to Neuromorphic Hardware: A Neural Network Model of Multisensory Integration

**DOI:** 10.3389/fnbot.2020.00029

**Published:** 2020-05-15

**Authors:** Timo Oess, Maximilian P. R. Löhr, Daniel Schmid, Marc O. Ernst, Heiko Neumann

**Affiliations:** ^1^Applied Cognitive Psychology, Institute of Psychology and Education, Ulm University, Ulm, Germany; ^2^Vision and Perception Science Lab, Institute of Neural Information Processing, Ulm University, Ulm, Germany

**Keywords:** multisensory integration, spiking neural network, neural network, neuromorphic processing, Bayesian inference, audio-visual integration, computational modeling

## Abstract

While interacting with the world our senses and nervous system are constantly challenged to identify the origin and coherence of sensory input signals of various intensities. This problem becomes apparent when stimuli from different modalities need to be combined, e.g., to find out whether an auditory stimulus and a visual stimulus belong to the same object. To cope with this problem, humans and most other animal species are equipped with complex neural circuits to enable fast and reliable combination of signals from various sensory organs. This multisensory integration starts in the brain stem to facilitate unconscious reflexes and continues on ascending pathways to cortical areas for further processing. To investigate the underlying mechanisms in detail, we developed a canonical neural network model for multisensory integration that resembles neurophysiological findings. For example, the model comprises multisensory integration neurons that receive excitatory and inhibitory inputs from unimodal auditory and visual neurons, respectively, as well as feedback from cortex. Such feedback projections facilitate multisensory response enhancement and lead to the commonly observed inverse effectiveness of neural activity in multisensory neurons. Two versions of the model are implemented, a rate-based neural network model for qualitative analysis and a variant that employs spiking neurons for deployment on a neuromorphic processing. This dual approach allows to create an evaluation environment with the ability to test model performances with real world inputs. As a platform for deployment we chose IBM's neurosynaptic chip TrueNorth. Behavioral studies in humans indicate that temporal and spatial offsets as well as reliability of stimuli are critical parameters for integrating signals from different modalities. The model reproduces such behavior in experiments with different sets of stimuli. In particular, model performance for stimuli with varying spatial offset is tested. In addition, we demonstrate that due to the emergent properties of network dynamics model performance is close to optimal Bayesian inference for integration of multimodal sensory signals. Furthermore, the implementation of the model on a neuromorphic processing chip enables a complete neuromorphic processing cascade from sensory perception to multisensory integration and the evaluation of model performance for real world inputs.

## 1. Introduction

While interacting with the world our senses are exposed to a rich and constant flow of information. Making sense of this vast of information is one of the most important task of our brain and crucial for survival. It does this by combing complementary information about the same event from different senses into a single percept. This integration process leads to an enhancement of the combined signal, thus supports the detection of events or objects of interest, improves disambiguation and allows for faster and more accurate processing than could be derived by a mere linear combination of unimodal information streams (Stein and Stanford, 2008).

Humans and other mammals are equipped with complex neural circuits to ensure fast, reliable and optimal combination of signals from various sensory organs (Marrocco and Li, 1977; Edwards et al., 1979; Cadusseau and Roger, 1985). This multisensory integration (MSI) process can be found already in the superior colliculus (SC) of the brain stem where auditory, visual and vestibular signals are combined to facilitate fast reflexive eye movements (Stein et al., 1983). This integration process is refined on ascending cortical pathways for higher level processing and decision making.

The SC is a melting pot of information from various sensory modalities and neurons in the SC are the first multimodal processing units in ascending sensory pathways (Meredith and Stein, 1983; Wallace and Stein, 1997) with spatially aligned receptive fields to these modalities (Meredith and Stein, 1996). The superficial layers of the SC receive mainly retinotopic inputs from the visual system and respond only to visual signals (Wallace et al., 1998). However, neurons in deeper layers of the SC gradually receive ascending inputs from other modalities and exhibit receptive fields for these modalities. In addition, their responses are multi-modal, i.e., receiving input from two different modalities leads to response characteristics that are different than responses to uni-modal signals (Stein and Stanford, 2008). Inputs to neurons in deep layers come from a diverse set of sensory systems and range from auditory signals from the inferior colliculus to proprioceptive signals from the vestibular system. To create a common frame of references for these different signals and, thus, spatially align them the retinotopic visual input is used as a guidance signal. This has been demonstrated in neurophysiological studies as well as modeling investigations (Rees, 1996; Wallace et al., 2004; Oess et al., 2020a).

Despite of all the ascending sensory signals in the SC, neurophysiological studies in cats indicate that there are several descending projections from the association areas (AES) of the cortex. Unimodal cortical projections from anterior ectosylvian visual area (AEv) and the auditory field of the anterior ectosylvian region (FAEs) are observed (Meredith and Clemo, 1989; Wallace et al., 1993; Wallace and Stein, 1994). These projections seem to play an essential role for the integration ability of SC neurons. Studies demonstrate that when these projections are deactivated, the neurons in the SC loose all their multisensory response characteristics (Alvarado et al., 2007a). These characteristics of SC neurons are complex and are the result of not just descending cortical projections but also neural circuitry and dynamics in the SC as we will describe later.

One of such a response characteristic is the so called *multisensory enhancement* which describes an enhanced activity for multisensory input signals that is higher than the linear combination of all unisensory inputs (Stein and Stanford, 2008). Such multisensory enhancement changes with the intensities of the input signals and creates the commonly observed and described *inverse effectiveness* for multi-modal signals of MSI neurons (Perrault et al., 2003; Stein and Stanford, 2008). That is, low intensity multimodal stimuli in spatial and temporal register lead to an enhanced response of MSI neurons which is greater than the summed responses for separately presented unimodal stimuli (super-additivity). In contrast, for high intensity multimodal stimuli, responses tend to be smaller than the sum of unimodal responses (sub-additivity). As a consequence, the probability of detecting low intensity events registered by two or more senses is increased.

Another important response characteristic of MSI neurons is the suppression for bimodal signals outside the receptive field of the neuron (Meredith and Stein, 1996). That is, the otherwise strong activity of SC neurons is suppressed by input signals of another modality with spatial or temporal offsets (spatial and temporal principle of multisensory integration). This suppression leads to a sub-additive combination of the two stimuli and thus can be seen as a means to prevent fusion of input stimuli that do not belong to the same event.

All these response characteristics can only be observed for active descending cortical feedback from association areas to MSI neurons in the SC (Jiang et al., 2001, 2007; Jiang and Stein, 2003; Alvarado et al., 2007a, 2009). When cortical projections or corresponding cortical areas are deactivated, multimodal response characteristics vanish (Rowland et al., 2014; Yu et al., 2016). Thereby, AES cortical feedback projections mediate multisensory integration abilities in SC neurons.

The aim of such a complicated integration process is to infer a percept that is more reliable and robust than unimodal perceptions. In fact, studies pointed out that humans integrate signals from different modalities in a statistically optimal fashion (Ernst and Banks, 2002), so called *Bayes optimal*. That is, they weight each signal based on its reliability before linearly combining them. Thereby, increasing the certainty of the combined signal. In addition, it has been suggested that in order to integrate signals in such an optimal way, neural populations need to be able to encode and integrate sensory signals Bayes optimally (Deneve et al., 2001; Ma et al., 2006). Hence, one of the challenges in computational modeling of multisensory integration is to demonstrate that a model integrates its input signals in a Bayes optimal or at least near-optimal way and to explain how the variety of response characteristics can emerge from population dynamics.

We introduce a neural network model of multisensory integration of audio-visual signals that exhibits such near-optimal Bayesian behavior, incorporates cortical feedback and demonstrates typical multi-sensory response characteristics. The contribution of this work is several-fold: First, we introduce a neural network model of conductance-based neurons in the superior colliculus that incorporates neurophysiological plausible cortical feedback connections. We investigate how this feedback alters the responses of multisensory neurons and enables them to integrate signals from multiple sensory streams. In addition, we examine what enables this process to integrate multimodal signals in a Bayesian optimal fashion and demonstrate that the introduced model does near-optimal Bayesian inference. This finding links the algorithmic mechanisms and representations to functionality. In a second part, we incorporate a spike-based output encoding of the model and deploy it on IBM TrueNorth neurosynaptic system (Cassidy et al., 2014), a neuromorphic processing chip with connections to neuromorphic sensory systems. Evaluations with this neuromorphic model demonstrate the performance for real world input data. This is a novel approach of testing a biological inspired architecture since it enables a complete neuromorphic processing cascade from sensory perception to multisensory integration and the evaluation of model performance for real world inputs.

## 2. Materials and Methods

In this section we introduce the architecture of the neural network model which interactions and components are based on physiological findings. The first part describes a rate-based model implementation with first-order ordinary differential equations defining the change of a neuron's voltage based activation. In a second part we introduce a spiking neural network model implementation and describe how it is realized on the TrueNorth neurosynaptic chip.

**Figure 1:**
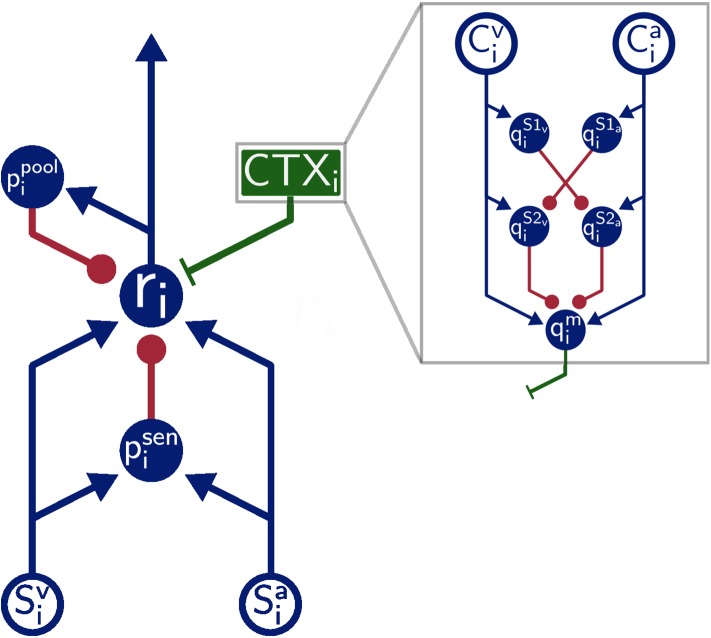
Model Architecture. Overall architecture of the multisensory integration model. Blue lines indicate excitatory connections, green lines indicate modulatory connections, and red lines indicate inhibitory connections. Green box is modulatory cortical signal that is describe in more detail in gray box. Filled circles represent model neurons, hollow circles indicate inputs to the model. Letters indicate the name of neurons and inputs.

The overall architecture of the model is inspired by the SC of mammals (Figure 1). That is, SC neuron populations (*r*) receive two modality specific excitatory inputs from a visual (*S*^*v*^) and auditory (*S*^*a*^) input population, respectively. Divisive inhibition of model neurons simulated by pool neurons (*p*^*pool*^) and feedforward inhibition of the sensory inputs ensure a normalized level of activity. This is realized via a coincidence detection mechanism of the feedforward inhibitory neurons (*p*^*sen*^). Thereby, these neurons provide inhibitory input to SC neurons only when bimodal sensory inputs are present but remains inactive for unimodal sensory inputs. Modulatory input from the cortex (green box CTX in Figure 1) facilitates multisensory integration abilities of the neurons by feedback top-down signals. We simulate this by a population of modulatory interneurons *q*^*m*^. Interactions in the cortex (gray box in Figure 1) are modeled such that they produce such signals only when both modalities (*C*^*v*^ and *C*^*a*^) receive inputs. This is achieved via a feedforward cross-inhibition in the feedback path of the cortical projections (gray box in Figure 1) between auditory and visual sensory areas (here $q_{a}^{S1}$, $q_{v}^{S1}$, $q_{a}^{S2}$, and $q_{v}^{S2}$). We will explain this in more detail in the next subsection.

### 2.1. Rate-Based Model

The rate-based model comprises several populations of neurons with dynamical interactions that together facilitate multisensory integration characteristics of SC neurons. Each of these populations comprises an array of *N* = 20 neurons, each one selective to a specific spatial location *i* in azimuthal direction (the center of the receptive field of the neuron). Such a count of neurons in a population is sufficient to achieve a satisfying resolution of the input space, which is arbitrarily chosen to be between 0 and 20 for the rate-based model. The number of neurons is not crucial and the model works well with larger neuron populations (>40) as well as smaller (<10) The membrane potential (as described in Equation 2) is governed by conductance-based integration of the neuron's excitatory and inhibitory inputs. Synapses are not modeled individually, but only represented in weight kernels which collect activities from pools of neurons. The inputs to such a neuron are described by $S_{i}^{a}$ and $S_{i}^{v}$, for auditory and visual inputs, respectively. They represent the activity of tonotopical visual neurons in superficial layers of the SC and auditory neurons in the external nucleus of the inferior colliculus (ICx) (Oliver and Huerta, 1992) that have presumably spatially ordered connections to the SC (Hyde and Knudsen, 2000, 2002; Knudsen, 2002). All inputs are assumed to be spatially aligned so that for a combined event of audio and visual signals at location *i*, SC neuron at location *i* receives the most activity from inputs $S_{i}^{a}$ and $S_{i}^{v}$. In addition, cortical activity of the AEv and FAEs is described with $C_{i}^{a}$ and $C_{i}^{v}$ and have the same activity characteristics as $S_{i}^{a}$ and $S_{i}^{v}$, respectively.

Inputs are assumed to be of Gaussian shape with uncertainty σ_*z*_ and intensity *I*_*z*_ (where *z* is *s*_*a*_ sensory auditory input, *s*_*v*_ sensory visual input, *c*_*a*_ cortical auditory signal or *c*_*v*_ cortical visual signal). Thus, auditory and visual inputs at location *i* can be described by

(1) $$\begin{array}{r} y_{i}\left(x_{t}\right)=\exp\left(\frac{-{\left(i-x_{t}\right)}^{2}}{2\cdot \sigma_{z}^{2}}\right)\cdot I_{z}, \end{array}$$

where *x*_*t*_ is the location of a stimulus at time *t*.

The core of the model is a population of multisensory SC neurons that integrates excitatory, inhibitory and modulatory inputs. The change of membrane potential *r*_*i*_ of an SC neuron selective to location *i* is described by

(2) $$\begin{array}{r} \tau_{d}{ṙ}_{i}=-\alpha_{d}r_{i}+\left(\beta_{d}-r_{i}\right)\cdot {EX}_{i}\cdot \left(1+\lambda \cdot {MOD}_{i}\right)-\kappa_{r}\cdot r_{i}\cdot {INH}_{i}, \end{array}$$

where parameter τ_*d*_ defines membrane time constant, α_*d*_ is a passive membrane leakage rate, β_*d*_ describes a saturation level of excitatory inputs and κ_*r*_ defines the strength of divisive inhibition. Parameter values are given in Table 1. The parameter λ defines the influence of the modulatory input, thus the multisensory enhancement strength of the model neuron. The firing rate of an SC neuron is calculated by a sigmoidal activation function *h* of its membrane potential *r*_*i*_

(3) $$\begin{array}{r} h\left(r_{i}\right)=\frac{2}{\left(1+exp\left(-{\left(r_{i}\cdot 3.4\right)}^{2}\right)\right)}-1. \end{array}$$

Excitatory inputs at location *i* are summarized in the term *EX*_*i*_, modulatory inputs from cortical projections are described by the term *MOD*_*i*_ and inhibitory inputs are summarized in the term *INH*_*i*_. We will describe each of these terms separately in the following.

**Table 1:** Model parameters.

**General parameters**			
N (# neurons)	20		
τ_*d*_	1.0	α_*d*_	1.0
β_*d*_	1.0		
σ^*m*^	3.0	σ	1.0
**SC neuron**			
κ_*r*_	0.25	λ	0.4
*l*	3.6		
**Modulatory neuron**			
κ_*m*_	1	γ_*m*_	5.0
β_*m*_	2.0		
**S2 cortical neuron**			
κ_*S*2_	1.0	γ_*S*2_	5.0

The excitatory input to SC neurons directly arises from visual and auditory neurons in the outer layers of the SC and ICx, respectively with spatially aligned receptive fields

(4) $$\begin{array}{r} {EX}_{i}\left(t\right)=S_{i}^{a}\left(t\right)+S_{i}^{v}\left(t\right). \end{array}$$

The modulatory input to SC neurons originates in the cortex and is defined by

(5) $$\begin{array}{r} {MOD}_{i}\left(t\right)=\underset{j}{\sum }\Lambda_{ij}^{m}\cdot g\left(q_{j}^{m}\left(t\right)\right), \end{array}$$

where $\Lambda_{ij}^{m}$ defines the interaction kernel of modulatory cortical projections to SC neurons and $g\left(q_{j}^{M}\left(t\right)\right)$ defines the activity of model neuron $q_{j}^{m}\left(t\right)$ at location *j* and time *t*. We define this neuron later in this section.

The inhibitory input to SC neurons comprises a signal from feedforward inhibitory neurons of sensory inputs and a self-inhibition neuron that is fed by a pool of integration neurons. It is defined by

(6) $$\begin{array}{r} {INH}_{i}=\underset{j}{\sum }\Lambda_{ij}\cdot g\left(p_{j}^{pool}\right)+\underset{j}{\sum }\Lambda_{ij}\cdot g^{sen}\left(p_{j}^{sen}\right) \end{array}$$

where $g\left(p_{j}^{pool}\right)$ is the activity of a modeled self-inhibitory neuron $p_{j}^{pool}$ and $g\left(p_{j}^{sen}\right)$ the activity of an inhibitory neuron $p_{j}^{sen}$ of feedforward inputs.

The feedforward inhibition of sensory inputs ensures similar intensity levels for unimodal and multimodal inputs by a coincidence integration of both inputs. That is, the feedforward inhibition inhibits SC neurons for simultaneously active bimodal inputs but not for unimodal inputs. Each SC neuron has a feedforward inhibitory neuron driven by spatially aligned auditory and visual inputs. The activation of the membrane potential for such a neuron is defined by

(7) $$\begin{array}{r} \tau_{sen}{ṗ}_{i}^{sen}=-\alpha_{sen}p_{i}^{sen}+\left(\beta_{d}-p_{i}^{sen}\right)\cdot S_{i}^{a}\cdot S_{i}^{v} \end{array}$$

Due to the multiplication of the neuron inputs, the feedforward inhibition neuron is active only if there are spatially aligned inputs of both modalities. Thus, the neuron behaves like a coincidence detector of its inputs.

A population of interneurons is modeled to realize pool normalization of SC neurons. A pool neuron is driven by neighboring SC neurons and feeds back on those SC neurons via inhibitory connections. The membrane potential of pool neurons is described by

(8) $$\begin{array}{r} \tau_{d}{ṗ}_{i}^{pool}=-\alpha_{d}p_{i}^{pool}+\left(\beta_{d}-p_{i}^{pool}\right)\cdot \underset{j}{\sum }\Lambda_{ij}\cdot h\left(r_{j}\left(t\right)\right). \end{array}$$

Together with the feedforward inhibition, the pool inhibition of model SC neurons serves as a normalization mechanism to ensure a normalized energy level over different input intensity levels.

Modulatory inputs that facilitate multisensory response characteristics have their origin in the cortex, namely in the association areas AEv and FAEs.

The membrane potential of such neurons is modeled by

(9) $$\begin{array}{rc} \tau_{d}{\overset{∙}{q}}_{i}^{m} & =-\alpha_{d}q_{i}^{m}+\left(\beta_{m}-q_{i}^{m}\right)\cdot \left(C_{i}^{a}+C_{i}^{v}\right)-\left(\gamma_{m}+\kappa_{m}\cdot q_{i}^{m}\right)\\ \cdot \underset{j}{\sum }\Lambda_{ij}\cdot \left(g\left(q_{j}^{S2_{v}}\right)+g\left(q_{j}^{S2_{a}}\right)\right), \end{array}$$

where parameter γ_*m*_ defines the subtractive influence of the inhibitory inputs $q_{j}^{S2_{a}}$ and $q_{j}^{S2_{v}}$ originating from a cross-modal inhibition circuit. This circuit (upper right part of gray box in Figure 1) ensures that only when both cortical inputs are present a modulatory signal is generated and fed back to SC neurons. If only one modality input is present the circuit is activated and generates strong inhibitory inputs for the *q*^*m*^ population resulting in a suppressed response of it. The circuit comprises four populations of neurons $q^{S2_{a}}$, $q^{S2_{v}}$, $q^{S1_{a}}$, and $q^{S1_{v}}$ with connections as shown in Figure 1 and membrane state equations for the auditory modality

(10) $$\begin{array}{r} \begin{array}{cc} \tau_{d}{\overset{∙}{q}}_{i}^{S2_{a}} & =-\alpha_{d}\cdot q_{i}^{S2_{a}}+\left(\beta_{d}-q_{i}^{S2_{a}}\right)\\ \cdot C_{i}^{a}-\left(\gamma_{S2}+q_{i}^{S2_{a}}\right)\cdot \underset{j}{\sum }\Lambda_{ij}\cdot g\left(q_{j}^{S1_{v}}\right),\\ \tau_{d}{\overset{∙}{q}}_{i}^{S1_{a}} & =-\alpha_{d}\cdot q_{i}^{S1_{a}}+\left(\beta_{d}-q_{i}^{S1_{a}}\right)\cdot C_{i}^{a}, \end{array} \end{array}$$

and visual modality

(11) $$\begin{array}{r} \begin{array}{cc} \tau_{d}{\overset{∙}{q}}_{i}^{S2_{v}} & =-\alpha_{d}\cdot q_{i}^{S2_{v}}+\left(\beta_{d}-q_{i}^{S2_{v}}\right)\\ \cdot C_{i}^{v}-\left(\gamma_{S2}+q_{i}^{S2_{v}}\right)\cdot \underset{j}{\sum }\Lambda_{ij}\cdot g\left(q_{j}^{S1_{a}}\right),\\ \tau_{d}{\overset{∙}{q}}_{i}^{S1_{v}} & =-\alpha_{d}\cdot q_{i}^{S1_{v}}+\left(\beta_{d}-q_{i}^{S1_{v}}\right)\cdot C_{i}^{v}, \end{array} \end{array}$$

Together these neurons synthesize the feedforward cross-modal inhibition circuit of cortical modulatory feedback.

The interaction kernel Λ between neuron populations is Gaussian and defined by :

(12) $$\begin{array}{r} \Lambda_{ij}=\frac{1}{\sigma \cdot \sqrt{2\pi }}\cdot \exp\left(-0.5\cdot {\left(\frac{i-j}{\sigma }\right)}^{2}\right) \end{array}$$

with σ = σ^*m*^ = 3 for Λ^*m*^ (the modulatory connection) and σ = 1 for Λ for all other connections.

The activation function *g*() for all neurons in the model except for neuron *r*_*i*_ to generate a firing rate from its membrane potential is a linear rectified function with saturation level of 1

(13) $$\begin{array}{r} g\left(x\right)=\left\{ \begin{array}{cc} 0, & \text{if}x<0,\\ 1, & \text{if}x>1,\\ x\cdot k, & \text{else}, \end{array}\right. \end{array}$$

with *k* = 2 for *p*_*sen*_ input and *k* = 1 otherwise.

### 2.2. Spike-Based Model

We also implemented the proposed MSI model on the IBM TrueNorth Neurosynaptic System. TrueNorth is a highly efficient, spiking, neuromorphic hardware (Merolla et al., 2014) that provides a million neurons and 256 million synapses organized into 4096 cores (Cassidy et al., 2014). This platform has been demonstrated in numerous real-time applications ranging from speech recognition (Tsai et al., 2017) over probabilistic inference (Ahmed et al., 2016) to motion detection (Haessig et al., 2018).

When transferring any rate-based model to a spike-based architecture one must choose a representation of real valued rates. Common options range from the spike-rate of single or groups of neurons, population codes, order of spike times (Trappenberg, 2010; Kasabov, 2019) to the (inverse) time between spikes (Haessig et al., 2018). The proposed rate-based model needs to evaluate several products of variables (e.g., in Equation 2), so we choose spike-rate as representation of real valued activation. Here, multiplication is realized simply by a logical AND operation, each of which can be done by a single hardware neuron on the TrueNorth.

**Figure 2:**
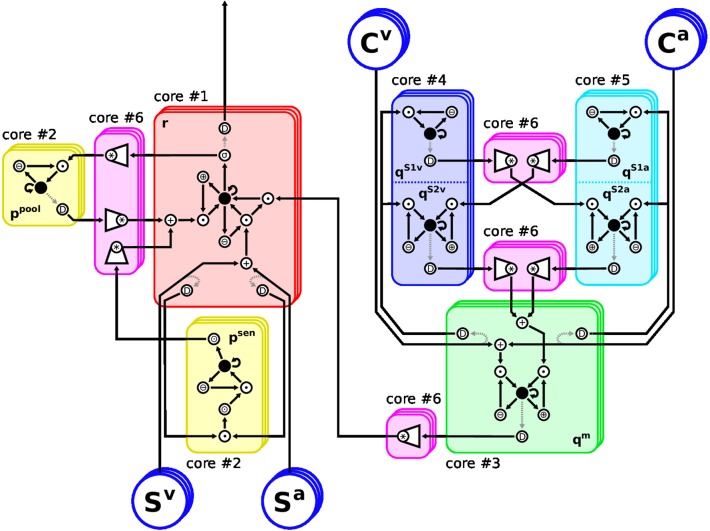
Arrangement of the Model Architecture on the TrueNorth neurosynpatic chip. Restrictions of the hardware require careful placement of neurons onto cores: The eight differential equations (ODEs) in Equations (2, 7–11) must be split into elementary operations, because they cannot be evaluated by a single neuron each. Also, when any neuron is required to deliver spikes to different cores, a splitter neuron must be inserted to duplicate their axon. Therefore, each feature channel consists of 60 hardware neurons instead of eight rate-based ones. The chosen layout realizes up to 21 feature channels of the proposed MSI model using six of the 4096 cores of TrueNorth. Stacked frames indicate channels. Neuron: ●, root neurons evaluating ODEs; *, convolution over channels; +/∙, weighted sum/product of two variables; ⊕/⊖/⊙, adding to/subtracting from/multiplying by a constant; D, splitter neurons; σ, sigmoidal transfer function. Rectified linear transfer functions are implemented using ⊙ and clipping.

The implementation follows our approach in Löhr et al. (2020): Any model equation is split into elementary operations, each of which can be handled by a hardware neuron. Such operations range from sums and products to non-linear functions. Neuronal activation is encoded directly as spike-rate, such that only values in [0,1] can be represented. Any exceeding value will be clipped, thus we must ensure that operational regimes of all dynamics lie in this range. Neurons with larger activation range are scaled down accordingly, and the weights of all their post-synaptic neurons are increased, respectively. Eventually, any differential equation in Equations (2, 7–11) is represented by a sub-graph of hardware neurons where the root neuron's activation follows said equation. The composition of these sub-graphs, forming the proposed model, is shown in Figure 2. The elementary functions to be performed by hardware neurons can be grouped into unary, binary and complex operations. *Unary* operations involve a constant and a variable, such as sum (⊕) and difference (⊖) or multiplication (⊙) by a factor. *Binary* operations involve two variables and examples are the weighted sum and product (+, •). *Complex* operations subsume all remaining neurons: Convolution over channels is done by weighted sum neurons (*), sharing afferent axons on the same core. Root neurons evaluating ODEs (●) and, finally, the sigmoidal (σ) activation function of Equation (3). The decomposition of equations into these elementary operations is shown in Table 2. For instructions on how to compute the TrueNorth neuron parameters of the above operations, please refer to the detailed Table 1 in Löhr et al. (2020).

**Table 2:** Elementary operations realized by hardware neurons.

**Operation**	**Usage**
⊕(const+var)	Inhib. terms $\left(\gamma_{m}+\kappa_{m}\cdot q_{i}^{m}\right)$, $\left(\gamma_{S2}+q_{i}^{S2_{a}}\right)$, $\left(\gamma_{S2}+q_{i}^{S2_{v}}\right)$ in Equations (9–11)
	Feedback term (1+λ·*MOD*_*i*_) in Equation (2)
⊖(const−var)	Conductance terms (β_*d*_−*r*_*i*_), $\left(\beta_{m}-q_{i}^{m}\right)$ in Equations (2, 9)
	and $\left(\beta_{d}-p_{i}^{sen}\right)$, $\left(\beta_{d}-p_{i}^{pool}\right)$ in Equations (7, 8)
	and $\left(\beta_{d}-q_{i}^{S1_{a}}\right)$, $\left(\beta_{d}-q_{i}^{S2_{a}}\right)$, $\left(\beta_{d}-q_{i}^{S1_{v}}\right)$, $\left(\beta_{d}-q_{i}^{S2_{v}}\right)$ in Equations (10, 11)
⊙(const•var)	Transfer func. *g*^*sen*^() used in Equation (6) and scaling of $S_{i}^{a}\cdot S_{i}^{v}$ in Equation (7)
+(var+var)	Components *EX*_*i*_ and *INH*_*i*_ of neuron *r* in Equations (4, 6)
	Excit. $\left(C_{i}^{a}+C_{i}^{v}\right)$ and inhib. $\left(g\left(q_{j}^{S2v}\right)+g\left(q_{j}^{S2a}\right)\right)$ inputs of *q*_*m*_ in Equation (9)
•(var•var)	Excitatory input $S_{i}^{a}\cdot S_{i}^{v}$ of *p*^*sen*^ in Equation (7)
	Products with conduct. terms (… )·*EX*_*i*_, $\left(\ldots \;\right)\cdot \left(C_{i}^{a}+C_{i}^{v}\right)$ in Equations (2, 9)
	and $\left(\ldots \;\right)\cdot \left(S_{i}^{a}+S_{i}^{v}\right)$, $\left(\ldots \;\right)\cdot \underset{j}{\sum }\ldots \;$ in Equations (7, 8)
	and $\left(\ldots \;\right)\cdot C_{i}^{a}$, $\left(\ldots \;\right)\cdot C_{i}^{v}$ in Equations (10, 11)
	Products with inhib. terms (… )·*INH*_*i*_, $\left(\ldots \;\right)\cdot \left(\underset{j}{\sum }\ldots \;\right)$ in Equations (2, 9)
	and $\left(\ldots \;\right)\cdot \underset{j}{\sum }\ldots \;$ in Equations (10, 11)
	Product with feedback term *EX*_*i*_·(1+λ·*MOD*_*i*_) in Equation (2)
*convolution	Weighted sums $\underset{j}{\sum }\Lambda_{ij}\ldots \;$ in Equations (5, 6, 8–11)
●root neurons	ODE terms $\overset{∙}{r_{i}}=-\alpha_{d}r_{i}+\left(\ldots \;\right)-\left(\ldots \;\right)$, ${\overset{∙}{q_{i}}}^{m}=-\alpha_{d}q_{i}^{m}+\left(\ldots \;\right)-\left(\ldots \;\right)$
	and ${\overset{∙}{p_{i}}}^{sen}=-\alpha_{sen}p_{i}^{sen}+\left(\ldots \;\right)$, ${\overset{∙}{p_{i}}}^{pool}=-\alpha_{d}p_{i}^{pool}+\left(\ldots \;\right)$
	and ${\overset{∙}{q_{i}}}^{S1_{a}}=-\alpha_{d}q_{i}^{S1_{a}}+\left(\ldots \;\right)$, ${\overset{∙}{q_{i}}}^{S2_{a}}=-\alpha_{d}q_{i}^{S2_{a}}+\left(\ldots \;\right)-\left(\ldots \;\right)$
	and ${\overset{∙}{q_{i}}}^{S1_{v}}=-\alpha_{d}q_{i}^{S1_{v}}+\left(\ldots \;\right)$, ${\overset{∙}{q_{i}}}^{S2_{v}}=-\alpha_{d}q_{i}^{S2_{v}}+\left(\ldots \;\right)-\left(\ldots \;\right)$
σ sigmoid	*h*(*r*_*i*_) in Equation (3)

As TrueNorth cores have a capacity of 256 neurons each, the proposed architecture must be split over several cores if more than six feature channels are to be used. At the same time, any hardware neuron's axon can only be routed to a single core. If its response is required on different cores, splitter neurons must be inserted which duplicate the neuron's response to provide additional axons. To keep the diagram simple in Figure 2 these splitters are connected to the neurons they duplicate via dashed arrows. However, they actually share the exact same input connections and internal parameters as their originals and thus produce a perfect copy of their spike patterns.

Thus we divided the spike-based model into six functional blocks of similar neuron count, each assigned to a respective core. To reduce the amount of splitter neurons, no sub-graph of a root neuron was split over different cores. Likewise, neurons realizing a convolution were placed onto a common core, so they can share presynaptic axons. The final hardware implementation of the proposed MSI model consists of 60 neurons per feature channel. Restricted to six of the 4096 cores this scheme allows to synthesize the MSI model with up to 21 feature channels. If more cores are used, the amount of feature channels can readily be increased to 256; a limitation due to the convolution operation. Convolutions over larger numbers of axons would need to be split over multiple cores, however, this would require additional splitter neurons as some input axons would be needed on different cores.

## 3. Results

Simulation results of the rate-based and spike-based model implementations for multisensory integration demonstrate characteristic multisensory integration properties and indicate near-optimal Bayesian inference of bimodal inputs.

In a first section, functional properties of the rate-based model and the multisensory integration are examined. Multisensory neurons are defined by their typical response behavior for bimodal and unimodal inputs (as described in section 1). Hence, in a first experiment we demonstrate the rate-base model's response behavior to multi- and unimodal inputs and investigate how inputs with spatial offsets are integrated. In particular, we test the response behavior for different stimulus intensities.

It has been shown that humans integrate signals from various modalities in an optimal fashion (Ernst and Banks, 2002). Where this integration exactly takes place is still under investigation. Model results demonstrate that already sub-cortical regions like the SC could integrate signals in a near optimal way when provided with a cortical control signal. Therefore, after presenting the characteristic response properties of multisensory integration neurons, we test the model's ability to integrate bimodal signals in a Bayes optimal fashion.

Having demonstrated that the rate-based model integrates multisensory signals near-optimally, in the second section we investigate the spike-based model implemented on IBM's neurosynaptic chip TrueNorth. In a first experiment, we reproduce the multisensory integration response characteristics to validate the correct functioning of the spike-based model. In a last experiment, we present real world data to the model recorded with neuromorphic hardware and evaluate its ability to integrate these signals.

### 3.1. Rate-Based Model Simulations

All experiments in this section are conducted with the rate-based model of multisensory integration. Simulation results in the following are computed from responses of model SC neurons after presenting the stimuli for 4,000 time steps. This duration is sufficient for each neuron in the neuron population to dynamically converge to its equilibrium membrane potential of numerical integration of the state equations. We chose *Euler's method* with a step size of δ*t* = 0.001 for numerical integration. Model parameters for following simulations are chosen to fit a variety of neurophysiological experiments (Meredith and Stein, 1996; Stein and Stanford, 2008). In particular, we focused on the response characteristics of multisensory neurons, e.g., inverse effectiveness, spatial principle, and sampled the parameter space manually to achieve qualitatively similar results.

**Figure 3:**
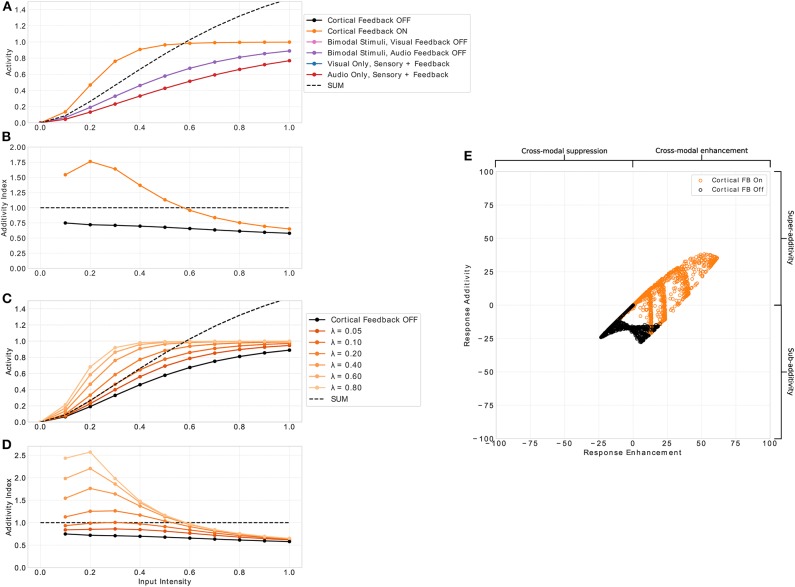
Multisensory enhancement of MSI neurons. **(A)** Displays neuron activity over input intensities. Black and orange lines indicate presence of both input modalities for sensory and cortical inputs (bimodal response). Orange lines indicates that cortical feedback is active whereas black lines shows responses when the feedback is turned off. Pink and purple lines indicate both sensory inputs present with cortical visual and audio input off, respectively. Blue and red lines show unimodal inputs that is only visual and auditory sensory and cortical inputs, respectively. Black dashed line is the sum of the unimodal inputs (red and blue lines). **(B)** Displays the additivity index over input intensities. It is calculated by the bimodal response divided by the sum of the two unimodal response strengths. Orange and black lines are the same conditions as in **(A)**. Plot **(C)** shows the additivity index over input intensities for several λ parameter values (fading orange lines) and cortical feedback projections off (black). **(D)** Displays the additivity index for the different values of λ. Right panel **(E)** displays a summary of responses taken from model neuron *i* = 8 of the spatial principle experiment (Figures 4, 7). The x-axis shows response enhancement of model neuron's responses. Positive values represent cross-modal enhancement whereas negative values indicate cross-modal suppression. The y-axis depicts the response additivity of the model neuron. Positive and negative values represent super- and sub-additivity, respectively. Orange dots indicate active cortical feedback projections. Black dots denote cortical feedback deactivated. Each dot corresponds to a certain input intensity, spatial offset value and randomly chosen value of σ_*s*_*a*__ and σ_*s*_*v*__ of input uncertainty in range [0.5, 5].

In all, we test 6 distinct stimulus conditions (see Figure 3): To demonstrate the importance of cortical feedback for multisensory integration, in the first condition (solid black line in following figures) all cortical inputs are absent (*C*^*a*^ = 0, *C*^*v*^ = 0), whereas both sensory inputs (*S*^*a*^, *S*^*v*^) are active according to Equation 1. The second condition (solid orange line) is the multisensory response for simultaneously active cortical and sensory inputs. In the third and fourth conditions (solid pink and purple line, respectively), both sensory inputs are present but only a single unimodal cortical input is given to demonstrate that both cortical unimodal signals are needed to facilitate multisensory integration. In the fifth and sixth conditions (solid blue and red lines, respectively), only unimodal sensory and unimodal cortical inputs (either visual or audio) are present as a control to show that the typical response characteristics emerge only for bimodal inputs which is in line with neurophysiological findings (Meredith and Stein, 1983; Meredith et al., 1992; Stein and Stanford, 2008).

#### 3.1.1. Inverse Effectiveness

An essential property of responses of SC neurons to multimodal inputs is the *inverse effectiveness* of stimulus intensity. That is, weak multimodal inputs create strong multisensory enhancement, whereas strong multimodal inputs only produce weak or no multisensory enhancement. To examine whether our model exhibits this response property we present spatially aligned auditory and visual inputs at location *i* to the model and measure the response of a representative neuron with receptive field centered at location *i*. Input intensities (*I*_*z*_ in Equation 1) are varied and a total of 11 intensities equally spaced in range [0, 1] are tested. The responses of the neuron as a function over intensities are depicted in Figure 3A. For input intensities lower than 0.55 the multisensory response is stronger than the sum of the responses to unimodal inputs. In contrast, for input intensities higher than 0.55 the neuron response is weaker than the sum. This property only emerges for conditions where both cortical inputs are present. If one or both of them are not present, the response of the model neuron is constantly below the sum of responses to unimodal sensory stimulation (purple and pink line). Thus, no multisensory enhancement takes place. The neuron parameter λ in Equation (2) controls the effect of the cortical projections and thus directly influences the multisensory enhancement of the neuron (see Figures 3C,D). This effect can be quantified by the *additivity index* which is defined as the ratio of the bimodal response to the sum of responses for unimodal inputs ($\frac{M}{V+A}$, where *M* is the multisensory response for active cortial projections, *V* unimodal visual response and *A* unimodal auditory response). An additivity index of 1 means the response for bimodal inputs is exactly as strong as the sum of both unimodal responses (see Meredith and Clemo, 1989 for details). Index values above 1 indicate super-additivity whereas index values below 1 indicate sub-additivity. The model neuron exhibits super-additivity for low input intensities and sub-additivity for high input intensities (see Figure 3B). Thereby, it exhibits inverse effectiveness response characteristics of multisensory neurons.

#### 3.1.2. Spatial Principle

**Figure 4:**
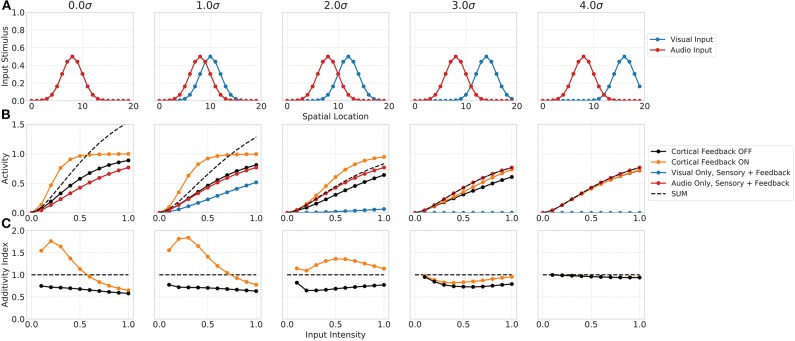
Spatial principle of MSI neurons. Upper row **(A)** displays the audio (red) and visual (blue) inputs to the model over spatial locations with increasing offset values. Middle row **(B)** shows the responses of rate-based model neuron *i* = 8 over input intensities for corresponding audio and visual inputs as given in the first row. Color code of lines is the same as in Figure 3. Bottom row **(C)** depicts the additivity index of the same model neuron *i* = 8 over input intensities.

The spatial principle of multisensory integration is commonly described by the inhibition of a stimulus in one modality by a stimulus of the other modality outside the receptive field of the neuron. Such a spatially separated stimulus combination not just leads to a reduction in the multisensory enhancement of the neuron but even to a suppression of its response. This suppression is usually ascribed to the Mexican hat shape of the receptive field. Our network model shows similar properties that emerge merely from the network dynamics (see Figure 4). The suppression of responses for spatially separated bimodal stimuli is facilitated by the feedforward inhibition of inputs in the network. In particular, the coupling of the absence of a spatial convolution kernel for excitatory inputs to SC neurons and the presence of such a spatial convolution for feedforward inhibitory inputs lead to a reduced activity of SC neurons outside the receptive field. The inhibition imposed by the spatially offset unimodal input still effects neighboring neurons whereas the direct excitatory does not. For bimodal stimuli with no spatial offset the network response is equal to the one shown in the previous experiment. However, for increasing spatial offset values (measured in σ of input Gaussian) the multisensory enhancement effect decreases (decreasing additivity index). For a stimulus with 3σ offset, the multisensory response is suppressed and lower than the unimodal response. This can also be seen in the additivity index curve that is below 1 at this offset. For offset values larger 3σ the suppression vanishes and the multisensory response is equal to the unimodal response, thus having an additivity index of 1.

#### 3.1.3. Interactions Among Within-Modality Inputs

**Figure 5:**
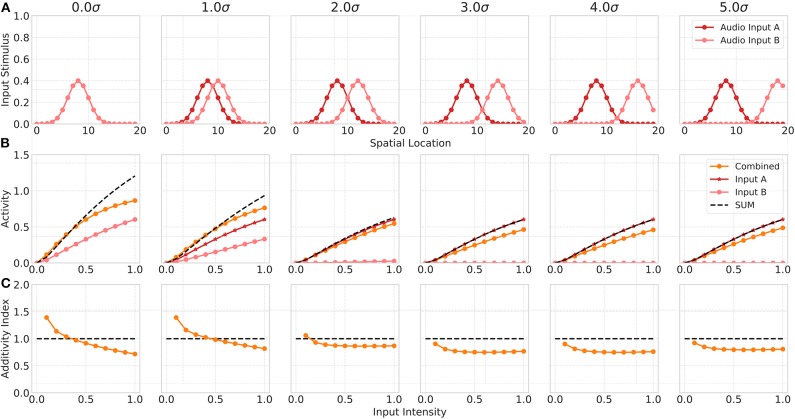
Within modality responses. Upper row **(A)** displays the audio A (red) and audio B (light red) inputs to the model over spatial locations with increasing offset values. Middle row **(B)** shows the responses of model neuron *i* = 8 over input intensities for corresponding unimodal inputs as given in the first row. Orange lines indicates combined response. Red line shows the response for audio input A, light red line for audio input B. Bottom row **(C)** depicts the additivity index of the same model neuron *i* = 8 over input intensities.

Neurophysiological studies have shown that multisensory neurons exhibit multisensory enhancement and inverse effectiveness only for bimodal inputs but not for multiple unimodal inputs (Alvarado et al., 2007b). In addition, the authors demonstrated in another study that the cortical projections only modulate multisensory but not unisensory integration (Alvarado et al., 2007a). Thus, in a third experiment we investigate model responses for two unimodal (auditory) inputs. In this simulation, we assume model input *S*^*v*^ to represent a second auditory stimulus (Audio Input B) and the visual cortical input *C*^*v*^ to be 0. We simulate two auditory inputs with activated auditory cortical projections but without visual cortical activity. Model responses for a combination of within-modal stimuli are higher than for a single unimodal input but show almost no super-additivity, expect for low intensity inputs (Figure 5). When one of the stimulus is moved outside the receptive field of the neuron, the combined response activity becomes lower than the response for a single unimodal input. This can be ascribed to a within-modal suppression (Alvarado et al., 2007b).

#### 3.1.4. Cortical Modulatory Projections

Cortical projections from association areas FAEs and AEv to the SC seem to have a crucial role in the multisensory integration behavior of MSI neurons and their response properties. For example, it has been shown that when these connections are removed or the corresponding cortical areas are deactivated, multisensory response properties in the SC vanish (Jiang et al., 2001; Alvarado et al., 2007a, 2009). We model these connections with modulatory input to the SC neuron that originates from a cross-modal inhibition circuit located in the cortex. That circuit ensures that only when a cross-modal stimulus is present the SC neuron receives modulatory inputs. Without such an input multisensory enhancement of SC neurons vanishes (see Figure 3B black line) and their response is very similar to responses for unisensory inputs. To investigate the role of cortical inputs in more detail we calculate the response enhancement (*M*−*max*(*V, A*))/(*M*+*max*(*V, A*)) that defines cross-modal enhancement and suppression for positive and negative values, respectively, and the response additivity (*M*−(*V*+*A*))/(*M*+(*V*+*A*))·100 that indicates super-additivity and sub-additivity for positive and negative values, respectively (see Avillac et al., 2007 for details). Only if the cortical inputs are active, multi-modal enhancement and super-additivity can be observed (see Figure 3E).

#### 3.1.5. Multisensory Inference

Several studies have shown that when multimodal sensory cues are simultaneously available, humans integrate these cues based on the reliability of each cue (Ernst and Banks, 2002). Thereby, human observers perform a weighted linear combination of cues from different sensory perceptions to maximize the certainty of the fused signal. The weight associated to a cue is proportional to the relative reliability of the perception of the corresponding cue. For example, estimating the size of an object by a combination visual and haptic sensory perceptions is usually based on the visual input. However, once visual input is blurred, thus the reliability of the perception is decreased, humans rely more on their haptic estimate (Ernst and Banks, 2002). By taking the reliability of the sensory perception as a weight in the integration process, humans perform optimal Bayesian inference for multisensory stimuli.

On which level in the processing hierarchy this inference takes place is not fully understood yet. Some researches argue that it is a rather high level cognitive process located in and between cortical areas (Kayser and Shams, 2015; Rohe and Noppeney, 2015). However, model simulations and neurophysiological recordings indicate that already on a level of two neural populations, Bayesian inference can take place (Ma and Pouget, 2008; Beck et al., 2012; Pouget et al., 2013). In our model we assume a combination of low level subcortical dynamics that provide a basis for cue integration together with high level cortical integration processes that facilitate subcortical near optimal multisensory integration.

**Figure 6:**
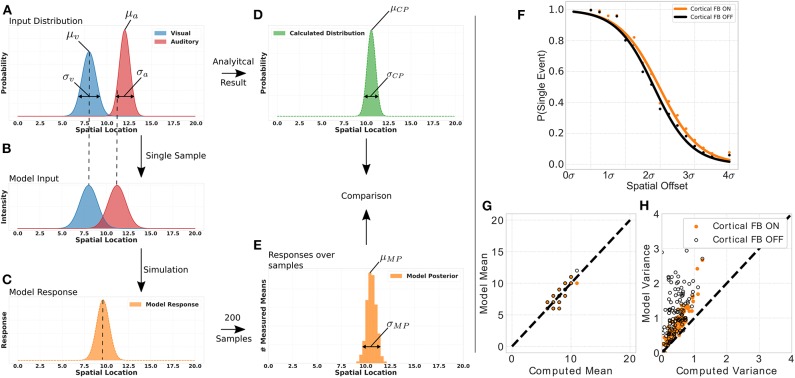
Bayesian inference experiment. Plot **(A)** displays the stimuli distributions for visual (blue) and audio (red) inputs, respectively, from which stimuli are sampled. Plot **(B)** represents an example of a single sample with stimuli for audio and visual inputs to the MSI neuron. Plot **(C)** depicts the model response for this single sample. The histogram in plot **(D)** is calculated analytically from the input distributions in plot **(A)**. The histogram in plot **(E)** is calculated from the model response over all samples. The probability of event fusion (single peak in model response) is given over spatial offset values in **(F)**. Dots represent mean values over several simulations with cortical feedback on (orange dots) and cortical feedback off (black dots). Sigmoid functions are fitted to the dots for better visualization. Active cortical feedback enables higher probability of event fusion over spatial offsets compared to inactive cortical feedback. Plot **(G)** compares the means of model responses vs. the analytically calculated means of the samples drawn as described in left panel. **(H)** Displays model variance values vs. computed variance values. Orange dots indicate active cortical feedback whereas black dots indicate cortical feedback off. Dashed line indicates perfect model behavior.

To demonstrate that this network structure allows our model to perform for near-optimal Bayesian inference we conduct a multisensory integration simulation experiment of auditory and visual inputs. For that, we define for each modality a normal distribution of stimuli location with a specific mean (auditory stimulus distribution's mean: μ_*a*_ = [8, 14.5], visual stimulus distribution's mean: μ_*v*_ = [5, 8]) and variance (auditory stimulus distribution's variance: σ_*a*_ = [0.5, 3.0], visual stimulus distribution's mean: σ_*a*_ = [0.5, 3.0]) and draw a sample location of a visual and auditory stimulus, respectively, from these distributions (Figure 6A). This location of audio and visual stimuli is then applied as input to the model, i.e., defines location *i* for a visual and auditory stimulus independently (Figure 6B). We independently draw a visual and auditory stimulus, respectively, 200 times from two distributions that have different mean and variance values. For each draw we present the two stimuli to the model, compute its responses and calculate the maximum of this response. We use only the maximum of the distribution to model a maximum-likelihood approach of choosing the stimulus location which has been previously observed in humans (Ernst and Banks, 2002). Taking together all maximum values over draws in a histogram results in a posterior distribution with a mean and variance value of model responses for a given stimuli set (see Figures 6C,E). The real posterior (see Figure 6D) of the combination of the two given distributions can be determined analytically with mean μ_*CP*_ = μ_*a*_·*w*_*a*_+μ_*v*_·*w*_*v*_, with $w_{a}=\frac{\frac{1}{\sigma_{a}^{2}}}{\frac{1}{\sigma_{a}^{2}}+\frac{1}{\sigma_{v}^{2}}}$ and $w_{v}=\frac{\frac{1}{\sigma_{v}^{2}}}{\frac{1}{\sigma_{a}^{2}}+\frac{1}{\sigma_{v}^{2}}}$, as well as variance $\sigma_{CP}=\frac{\sigma_{v}^{2}\cdot \sigma_{a}^{2}}{\sigma_{v}^{2}+\sigma_{a}^{2}}$.

We show the model's inference capability by comparing it's response with calculated mean and variance of the stimulus input (see Figures 6G,H). Two conditions are tested, cortical feedback on and off. For a fair comparison only model responses that show a single peak (fused responses) are considered. Model and analytical calculated mean are similar under both conditions. However, the cortical feedback allows for a more precise computation of the inferred variance. The variances of model responses without cortical feedback has a higher offset and increases dramatically for large input variances. In contrast, with cortical feedback this increment is smaller and there is almost no offset.

The nervous system can combine two events from different modalities to form a combined single percept of the event. Under what conditions such a *mandatory cue fusion* takes place has been investigated thoroughly (Hillis et al., 2002). One crucial factor for event fusion is the spatial discrepancy between the different modalities. If an auditory event origins from the same or similar location as a visual event, it is likely to be fused to a single event. However, when the spatial offset between the two events increases the likelihood of perceiving a single event decreases (Andersen et al., 2004; Stevenson and Wallace, 2013). We investigate this behavior for our model by calculating the percentage of samples where a single event (one peak in response, mandatory fusion) has been detected in contrast to samples where two events (multiple peaks in response, no fusion) are present (see Figure 6F). The probability of event fusion is constantly higher for activate cortical projections than without.

### 3.2. Spike-Based Model Simulations

**Figure 7:**
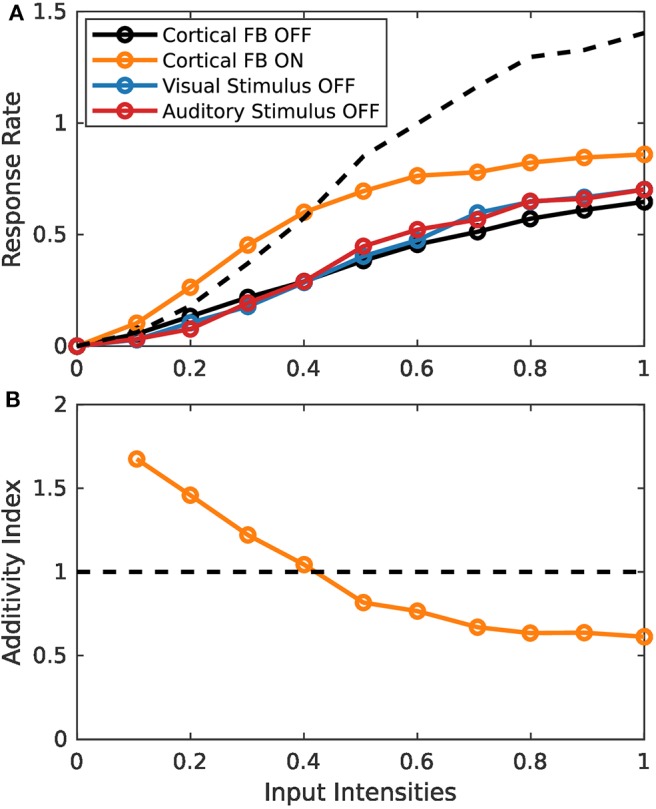
Inverse effectiveness of spiking MSI neuron implementation. **(A)** Displays spike-based neuron activity over input intensities. Black and orange lines indicate presence of both input modalities for sensory and cortical inputs (bimodal response). Color code as in Figure 3. **(B)** Displays the activity index over input intensities. It is calculated by the bimodal response divided by the sum of the two unimodal response strengths. Orange and black lines are the same conditions as in **(A)**.

Simulation experiments in this section are conducted with the spike-based model implementation on the TrueNorth neurosynaptic chip. Since implementation of the model on this chip is fundamentally more complex than the rate-based variant, we first demonstrate similar behavior of the two implementations by presenting the same stimulus set of increasing input intensities as for the rate-based model in section 3.1.1. Typical response characteristics of MSI model neurons can be observed for the spiking model implementation (Figure 7). For low intensity inputs the bimodal response is greater than the sum of the two unimodal inputs (super-additivity). For increasing intensities this enhancement is reduced until the bimodal response is lower than the sum of the two bimodal inputs (sub-additivity). Thereby, the spike-based model demonstrates inverse effectiveness of MSI neurons.

We conduct the following simulation experiment to demonstrate the model's ability to cope with real world event-based sensory input data.

**Figure 8:**
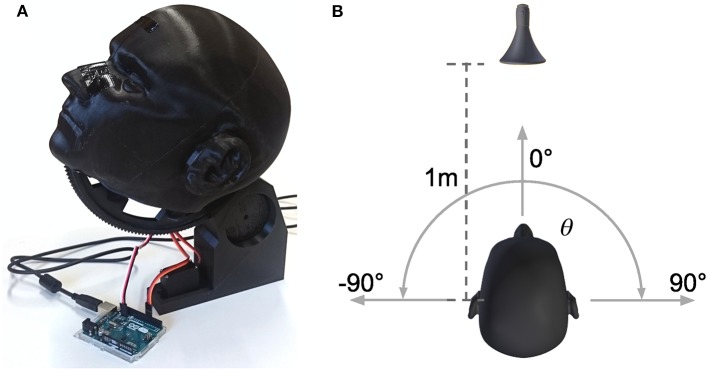
Device and setup for real world sound recordings. **(A)** Shows the 3D printed, anatomical correct replication of a human head and ears. In the ears, ~2 cm inside the ear canal, standard microphones are placed for recording. The head is controlled by two servo motors, one for turning around the longitudinal axis (azimuth), the other for turning around the frontal axis (elevation). All sounds are recorded for 0° elevation (head facing the speaker) and different azimuth directions. **(B)** Shows the setup for the recordings. The head is centered 1*m* in front of a standard speaker and can rotate around its longitudinal axis from θ = −90° to θ = 90° with a minimal step increment of 1°.

Inputs to the model are generated by a neuromorphic vision sensor (DVS) (Lichtsteiner et al., 2006) and an artificial neural implementation of functions of a cochlea. Sounds are recorded from 19 locations equally spaced in azimuthal range [−90, 90]° via two microphones placed inside the right and left ear canal of human-like shaped ears on a dummy head as depicted in Figure 8A. The device can turn around its longitudinal axis to create a relative displacement of the sound source location in the horizontal plane. The distance to the speaker (standard speaker) remains constant (1m) during movement (Figure 8B). We choose the sound of a vacuum cleaner for real world recordings. The presented sound type is a monaurally recorded sound of a vacuum cleaner. This sound was presented for azimuthal head directions of [−90, −70, −50, −30, −10, 10, 30, 50, 70, 90]° and 0° elevation. It was played back from the speaker and recorded in stereo with the two in-ear microphones for the duration of the sound. All recordings were done in a sound attenuated room. Subsequently, a bank of gammatone-filters is separately applied to these recordings of the right and left ear, thus creating spectrograms with 64 frequency channels resembling the output of the cochlea. Each spectro-temporal bin in the spectrogram is converted to a spike train and fed to a spiking neural network model of the lateral superior olivary (LSO) complex for computation of interaural level differences (ILD). Output of LSO model neurons is averaged over frequency bands. The weight channel of the left hemispherical output with maximum activity is subtracted from the weight channel of the right hemispherical output with maximum activity. This leads to a combined response of the left and right hemisphere over the entire range of perceived ILD values. Subsequently, this signal is converted to a one-dimensional estimate of spatial activations of sound sources by a set of 19 radial basis functions (RBFs). These functions are tuned to a specific response rate of LSO neurons, thus encoding a unique spatial location in range [−90°, +90°] in 10° steps from the rate of LSO neurons (see Oess et al., 2020b for a detailed description of this preprocessing).

Videos are recorded of a stationary tea cup placed at evenly spaced positions (range [−27, +27]*cm* in 3*cm* steps) in front of the camera (distance 177*cm*). The cup's positions in all videos combined span the complete horizontal field of view of the camera. To ensure that stationary contrasts are detected the mirror setup of Löhr and Neumann (2018) is used which adds random tremor to the DVS's optical axis. A Gaussian subsampling scheme reduces the visual input of 128 × 128 pixels to 1 × 19 neurons, which relate to azimuthal direction. These neurons resemble those in superficial layers of superior colliculus and directly serve as visual sensory inputs to the MSI model.

**Figure 9:**
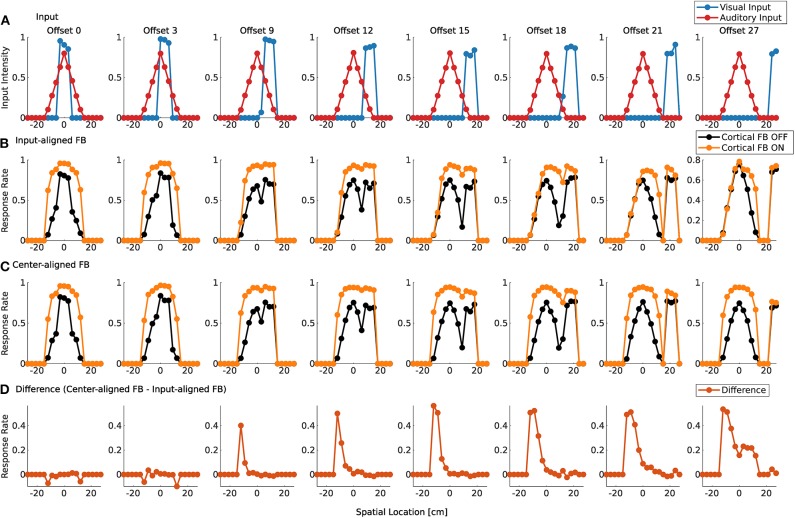
Spatial fusion for spiking MSI neurons. Upper row **(A)** displays the audio (red) and visual (blue) real world inputs to the model over spatial locations with increasing offset values. The inputs originate from LSO model and camera recordings. Offset is given in evenly spaced displacement of stimuli in cm. Row **(B)** shows multisensory response rates of spike-based model neurons over spatial locations for corresponding audio and visual inputs as given in the first row. Orange lines indicate cortical feedback. Black lines indicate cortical feedback off. Spatial locations are given in cm. Note that the offsets are not equally distributed in the figure due to spatial constraints. We choose to display only offsets for responses that are substantially different. Row **(C)** shows multisensory response rates of spike-based model neurons as in row **(B)**. However, in contrast to **(B)**, visual and auditory cortical feedback locations are fixed to the location of the auditory sensory stimulus (0 cm). The lower row **(D)** displays the difference between response rates in **(B,C)** for active cortical feedback projections.

Visual and auditory real world stimuli with increasing spatial offsets are presented to the model as sensory inputs. Audio and visual cortical inputs are simulated as described in Equation (1) and follow their corresponding sensory counterpart over spatial offsets. That is, their mean is set to the location of the maximum sensory response of the real world input. For small spatial offsets the two stimuli are integrated and a single peak in the model response is present (Figure 9A). We take this as an indication that multisensory fusion takes place and a single event is perceived. For increasing spatial offsets (>12 cm) the model response without feedback shows two separate peaks. This indicates that no integration takes place anymore and two separate events are perceived. The offset value for which this change of perception takes place changes with active cortical feedback projections (>18 cm). This demonstrates that cortical feedback facilitates larger offsets for which two stimuli are fused to a single percept. For an offset of >30 cm multisensory enhancement vanishes and responses for activated and deactivated cortical feedback projections are similar.

To test whether this reduction in multisensory enhancement is due to the spatial offset of sensory inputs or cortical inputs, the location of the cortical projections is fixed at the location of the auditory input (−9 cm) over all offset values. Thereby, only sensory inputs at this location receive modulatory cortical feedback (Figure 9C). The visual stimulus is shifted away from the auditory stimulus which leads to the perception of two different events for offset values larger than 12 cm (cortical feedback projections inactive) or 18 cm (cortical feedback projections active). This is similar to responses in Figure 9B. However, multisensory enhancement is maintained even when the visual input is shifted further away from this location. For offset value of 30 cm multisensory enhancement is still present at the location of the auditory stimulus whereas for the visual stimulus such an enhancement does not take place, as can be seen in Figure 9D.

## 4. Discussion

We introduced two implementations of a neural model simulating functions of SC neurons for integration of audio-visual signals. The model incorporates modulatory cortical feedback connections to facilitate enhancement of multisensory signals. The rate-based implementation of the model and its responses were evaluated in various simulation experiments and we demonstrated the importance of cortical feedback projections for near-optimal integration of signals. Furthermore, the spike-based model implementation on neuromorphic hardware showed its capability of integrating real world spike inputs from neuromorphic sensors.

### 4.1. Multisensory Integration

Typical multisensory neurons show response enhancement for multimodal stimuli that arrive in temporal and spatial coincidence (Meredith and Stein, 1996). Previous studies report that this property only arise for enabled cortical feedback projections (Stein et al., 1983; Wallace and Stein, 1994; Jiang et al., 2001; Alvarado et al., 2007b). Our model results replicate such observations and show that response enhancement can vary with the gain of the modulatory cortical feedback projections controlled by λ parameter (Equation 2) in the model (see Figures 3D,E). Thus, the gain of how neurons integrate modulatory feedback could explain the observed variety of multisensory enhancement in responses of SC neurons as has been observed previously (Kadunce et al., 2001). Without cortical projections the response to multisensory input remains sub-additive (see Figure 3B) even for high input intensities. Such cortical projections are only activated when both modality specific cortical signals are active. If only one cortical region is active multisensory response properties vanish. This is in line with findings in cats, where multisensory integration disappears for deactivated cortical areas (Meredith and Clemo, 1989; Alvarado et al., 2009). In our model, this is achieved with a specially designed cortical cross-modal forward inhibition circuit in the feedback projections (see Equation 9).

Furthermore, the model follows the previously described spatial principle of MSI neurons (Meredith and Stein, 1996) (see section 1 for definition) by suppressing responses for bimodal stimuli with large spatial offsets. We would like to point out that this suppression is achieve merely by dynamic interactions between the pool normalization, the feedforward inhibition and excitation of sensory neurons, thus implicitly creating a center surround receptive field of MSI neurons.

#### 4.1.1. Bayesian Inference

Several investigations show that afferent connections from cortical regions to the SC are necessary for multisensory integration (Alvarado et al., 2007a, 2008, 2009). However, the functional purpose of such feedback projections is still unclear. Our Simulation experiments show that multisensory integration of two input signals in a near-optimal Bayesian way appears only when cortical feedback projections are active. The variance of the integrated signal is substantially similar to the computed, optimal value for active projections than compared to responses without these projections (see Figure 6G). This is especially true for larger spatial offsets of the two input stimuli (see Figure 6F). Thus, we hypothesize that one purpose of cortico-collicular feedback is to facilitate optimal integration of multimodal signals and that such an optimal integration might already happen on the level of the SC. Presented model variance values (Figure 6H) exhibit an offset for higher variance values which we assume to result from the static size of the receptive field of model neurons and could be compensated with a dynamically changing receptive field depending on sensory certainty.

### 4.2. Neuromorphic Implementation

We demonstrated that the proposed model architecture is suitable for robotic applications by implementing it on a real-time neuromorphic processing chip. Preliminary results for real world spike recordings obtained by neuromorphic sensory hardware suggest that the model is robust and capable of integrating real world multisensory signals. It was shown that the model's ability to fuse two modalities into a single percept changes with cortical feedback projections. This supports the hypothesis that cortex plays a crucial role in determining whether two stimuli belong to the same event or if they represent two separate events. This is further investigated in a last experiment in which the cortical feedback signals are fixed to the location of the auditory sensory input while the visual sensory input is spatially shifted. The response enhancement remains at the auditory location even if the sensory visual input is not present anymore. This can be interpreted as an increased cortical focus for this specific location. Thus, cortical projections might be controlling the mandatory fusion range of multisensory neurons and in addition serve as a spatial attention signal, as has been suggested by McDonald et al. (2001); Mozolic et al. (2008), and Talsma et al. (2010).

In future experiments, we are planning to implement such a spatial attention mechanism in order to selectively choose which multisensory signals should be enhanced. We believe that this could be accomplished by a more sophisticated cortical feedback signal with spatial properties different than the perceived sensory inputs.

### 4.3. Comparison to Other Models for Multisensory Integration

Several models that account for multisensory integration in the colliculus of different granularity and focus have been suggested over the years. Some of them try to explain the various response properties of MSI neurons (Anastasio and Patton, 2003; Ursino et al., 2017) whereas others focus more on the biological detailed architecture (Cuppini et al., 2011, 2017; Casey et al., 2012). In the following, we will describe two of them and point out their strengths and weaknesses compared to our presented model.

In Rowland et al. (2007), the authors presented an algebraic and compartmental model of multisensory integration that incorporate cortico-collicular projections and try to explain the existence of AMPA and NMDA receptors in MSI neurons. Their goal was to reproduce a variety of physiological findings without paying much attention to the underlying biological anatomy and structure. Like our model, the authors are able to reproduce several MSI characteristics like multisensory enhancement, inverse effectiveness and super- and sub-additivity. In addition to our presented results, they also demonstrate the MSI neuron dependence on NMDA receptors and the temporal window of integration of their model. However, they did not present any results that indicates a Bayesian optimal integration of the signals.

Another approach is taken by Ohshiro et al. (2011) and their normalization model in which they show that many of the MSI response characteristics can be achieved by a pool normalization of the neuron output. Their model assumes MSI neurons that integrate signals according to a linear weighted sum with different input weights across modalities and neurons. In addition to the replication of MSI characteristics, the authors performed a virtual experiment of vestibular-visual integration task with their model and provided data that closely resembles findings in monkeys. Despite the profound analysis of their model and resemblance of experimental data, the authors neglect cortical projections to MSI neurons entirely.

### 4.4. Limitations of the Model

As we have shown, the two proposed model implementations using rate-based and spike-based encoding are both able to replicate several physiological findings, predict the purpose of cortical modulatory projections and are capable of reliably processing real world spiking data. One of the drawbacks of the current implementations is the lack of any learning mechanism in the process. The model assumes that all connections are already established and inputs are spatially aligned, even though, studies show that multisensory integration emerges during maturation of the nervous system by a constant exposure to multimodal signals (Wallace and Stein, 1997). This long term exposure influences how and to what extent multisensory integration takes place. This limitation in our model could be tackled by incorporating a Hebbian correlation learning mechanism between the cortical feedback projections and MSI neurons as well as the inputs of the model. The current assumption that the two modalities are spatially aligned is a strong constraint and simplifies the model architecture but is not biologically plausible. We are confident that this can be overcome with a previously proposed architecture of spatial map alignment of visual and auditory inputs (Oess et al., 2020a).

### 4.5. Outlook

The proposed model implementations of MSI neurons set a solid basis for future investigations. One important question we are planning to investigate is the role of the cortical feedback. One plausible hypothesis is that the feedback projections can be controlled by an attention mechanism to set special focus on a particular region and thereby enhances signals at that spatial location. This is an essential mechanism when conflicting events are present. In addition, the spike-based implementation of the model on neuromorphic hardware is an important step toward a real-time capable robotic platform. This platform will be equipped with audio and visual sensory hardware which directly communicates with the neuromorphic processing chips via spike trains, thereby creating a complete neuromorphic system from the sensory perception to decision making and action execution.

## Data Availability Statement

The datasets generated for this study are available on request to the corresponding author.

## Author Contributions

TO contributed in rate-based model conception and design, experiment conduction, analysis and interpretation of data, and drafting manuscript. ML contributed in spike-based model conception and realization and drafting the manuscript. DS contributed in spike-based model conception and realization and data interpretation and analysis. ME contributed in analysis and interpretation of data, and critical review. HN contributed in model conception and design, analysis and interpretation of data, and critical review.

## Conflict of Interest

The authors declare that the research was conducted in the absence of any commercial or financial relationships that could be construed as a potential conflict of interest.
